# Synthesis and Characterization of Novel Patchouli Essential Oil Loaded Starch-Based Hydrogel

**DOI:** 10.3390/gels8090536

**Published:** 2022-08-26

**Authors:** H. P. S. Abdul Khalil, Syaifullah Muhammad, Esam Bashir Yahya, Lee Kar Mun Amanda, Suriani Abu Bakar, C. K. Abdullah, Abd Rahim Aiman, M. Marwan, Samsul Rizal

**Affiliations:** 1 Bioresource Technology Division, School of Industrial Technology, Universiti Sains Malaysia, Penang 11800, Malaysia; 2 Nanotechnology Research Centre, Faculty of Science and Mathematics, Universiti Pendidikan Sultan Idris, Tanjong Malim, Perak 35900, Malaysia; 3 Chemical Engineering Department, Universitas Syiah Kuala, Banda Aceh 23111, Indonesia; 4 ARC-PUIPT Nilam Aceh, Universitas Syiah Kuala, Banda Aceh 23111, Indonesia; 5 Bioprocess Technology Division, School of Industrial Technology, Universiti Sains Malaysia, Penang 11800, Malaysia; 6 Green Biopolymer, Coatings and Packaging Cluster, School of Industrial Technology, Universiti Sains Malaysia, Penang 11800, Malaysia; 7 School of Biological Sciences, Universiti Sains Malaysia, Penang 11800, Malaysia; 8 Department of Mechanical Engineering, Universitas Syiah Kuala, Banda Aceh 23111, Indonesia

**Keywords:** hydrogel, waxy starch, patchouli essential oil, antibacterial, viscosity, biocompatibility

## Abstract

Starch hydrogels are highly available, biocompatible and biodegradable materials that have promising applications in medical and pharmaceutical industries. However, their applications are very limited due to their poor mechanical properties and fragility. Here, we investigated, for the first time, conventional corn and waxy corn starch-based hydrogels for loading patchouli essential oil. The essential oil extracted by supercritical carbon dioxide with a yield reached 8.37 ± 1.2 wt.% (wet sample) at 80 °C temperature and 10 MPa pressure. Patchouli essential oil exhibited a 23 to 28 mm zone of inhibition against gram-positive and gram-negative bacteria. Waxy starch hydrogels had better properties in term of viscosity, water evaporation stability and the delivery of essential oil than conventional starch hydrogels. The viscosity and spreadability of a 6% waxy starch sample were 15,016 ± 59 cP and 4.02 ± 0.34 g·cm/s, respectively, compared with those of conventional starch hydrogel (13,008 ± 29 cP and 4.59 ± 0.88 g·cm/s). Waxy starch-based hydrogels also provided slower in vitro biodegradation behavior and sustained release of essential oil compared with conventional starch hydrogels. All the samples were biocompatible and non-cytotoxic to fibroblast cells; the addition of patchouli essential oil enhances the proliferation of the cells. The enhanced viscosity, good antibacterial and improved biocompatibility results of prepared hydrogels confirm their suitability for wound healing applications.

## 1. Introduction

Hydrogel is a material resulting from physical and/or chemical cross-linking of the same or between different materials’ macromolecules, resulting in a 3D network capable of retaining different amounts of water without disintegrating [1,2]. Owing to their hydrated system, they are ideal carriers for drugs and bioactive compounds [3,4]. Synthetic polymer-based hydrogels such as polyvinyl alcohol, polyamides, carbopol and polyethylene glycol possess several interesting characteristics including high mechanical properties and strong stability against disintegration [5]. However, the potential toxicity, non-biodegradability and non-biocompatibility of such materials have restricted their usage, and most researchers nowadays are looking for bio-based alternatives. Although several biopolymers have been used in hydrogel preparation, including chitosan, alginates and starch, due to their unique properties, these natural materials have weaker mechanical properties and require further modification to compete with the synthetic-based hydrogels.

Recently, many works have been carried out on developing starch-based hydrogels from potato, corn and even soluble starches [6]. The fabrication process involves the gelatinization of starch to form a 3D network to disrupt the starch crystalline structures and facilitate biopolymer chain–chain interaction. This approach involves solubilization of starch powder and heating in water [7,8]. However, starch hydrogel can also be gelatinized by using the chemical route through an alkaline solution. Waxy starch is a type of starch exclusively consisting of amylopectin with a higher molecular weight and higher branching than the conventional starch. Hsieh et al. [9] reported that waxy starch typically had greater paste viscosities than the normal starch, making it preferable in hydrogel formation. Unlike waxy starch, normal starch contains both amylose and amylopectin. It is difficult to define the exact role of amylose and amylopectin in hydrogel formation and their role in encapsulation or delivery of other materials, such as essential oils. To the best of our knowledge, until now, the effect of normal and waxy starch hydrogel in the delivery of essential oils is yet to be determined.

Patchouli (*Pogostemon cablin*) is an aromatic flowering plant in the family Lamiaceae, which is commonly known as the mint or deadnettle family [10]. Its essential oil is widely utilized in several industries, including perfumery and cosmetic products such as deodorants, soaps and detergents [11]. Furthermore, patchouli essential oil has a variety of pharmacological and medicinal activities, including antimicrobial, anti-inflammatory and anti-cancer effects, in addition to improving gastrointestinal function and enhancing memory [12]. This essential oil is conventionally obtained by steam distillation of the plant biomass that includes non-fermented dried leaves. However, patchouli essential oil, as with most essential oils, exhibits a volatile nature, making it sensitive to high temperature, which may affect its pharmaceutical properties. In this study, we used supercritical carbon dioxide with mild temperature for the extraction of patchouli essential oil to avoid any possible denaturation of its active compounds. The extracted essential oil was loaded for the first time in starch–Carbopol hydrogels. Two different starches were used: the conventional corn starch and waxy starch, to investigate their viscosity and spreadability properties and the release of the essential oil.

## 2. Results and Discussion

### 2.1. Extraction and Characterization of Patchouli Essential Oil

**Figure 1:**
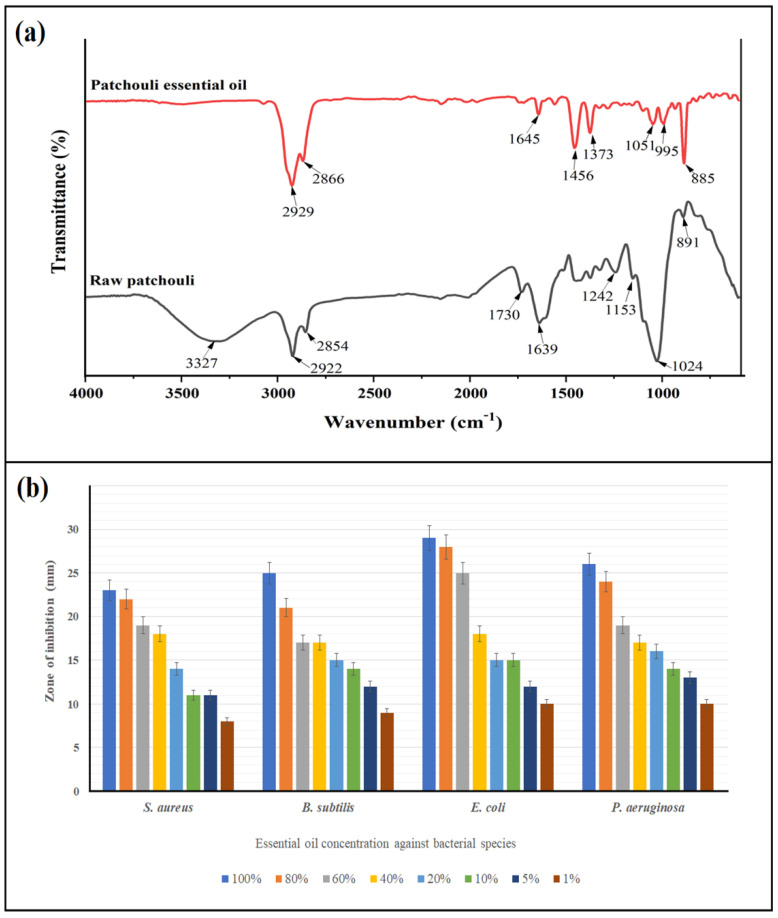
Characterization of patchouli essential oil; (**a**) FT-IR spectra and (**b**) antibacterial activity against the selected microorganisms.

The extraction of patchouli essential oil was carried out by using a supercritical carbon dioxide approach, with the yield reaching 8.37 ± 1.2 wt.% (wet sample) under the conditions of 80 °C temperature and 10 MPa pressure. FT-IR spectra of the raw patchouli plant (leaves) and its extracted essential oil are presented in Figure 1a. Generally, more than 20 chemical components are often present in patchouli essential oil, although some studies have reported up to 50 chemical compounds [10]. The vibrations in raw patchouli at 3327 cm^−1^ correspond to the OH stretching, which is absent in the essential oil due to the purity and high concentration of the prepared essential oil. Two bands can be clearly seen at 2922 and 2854 cm^−1^, which slightly shaft to 2929 and 2866 in the essential oil, and correspond to methyl (–CH_3_) stretching vibration (asymmetric and symmetric) [13]. C=O carbonyl aldehyde at 1730 cm^−1^ also shafted to 1645 cm^−1^; the presence of this vibratory stretching confirms the high amount of aldehyde compounds in patchouli [14]. A peak at 1445 cm^−1^, only present in the essential oil, corresponds to a stretching of C-H bond and C=C stretching of the alkane and aromatic ring, respectively. Other peaks can also be found at the range of 1051 to 885 cm^−1^, which indicates other functional groups including ether (C–O) and acetylene (–HC=CH–) [15].

Patchouli essential oil also exhibited strong antibacterial activity against gram-positive (*Staphylococcus aureus*, *Bacillus subtilis*) and gram-negative bacteria (*Escherichia coli*, and *Pseudomonas aeruginosa*.). Figure 1b presents the results of antibacterial activity of different concentrations of patchouli essential oil. It can be seen that gram-negative bacteria was more sensitive to the oil, compared with the gram-positive ones, which can be attributed to the thick layers’ peptidoglycan, that caused difficulties for the oil penetration to the bacterial cells. Gram-negative bacteria is known for its thin layers of peptidoglycan [16], thus, *E. coli* was the most sensitive bacteria to the essential oil with an inhibition zone of 29 mm, followed by *P. aeruginosa* (26 mm). Surprisingly, S. aureus was the most resistant bacteria, with an inhibition zone of only 23 mm. Typically, the antibacterial activity of the essential oil decreased with the decrease in the oil concentration. The MIC and MBC varied among the bacterial species: *E. coli* and *P. aeruginosa* had a similar MIC of 3 mg/mL but different MBC (3 mg/mL for *E. coli* and 4 mg/mL for *P. aeruginosa*), which is lower than that achieved by Yang et al. [17], who reported 4 and 5.5 mg/mL MIC and MBC of 2 and 10 mg/mL for *E. coli* and *P. aeruginosa*, respectively. Owing to the presence of several active compounds in the essential oil, broad antimicrobial activity of patchouli was also confirmed by Thoppil et al. [18]. Our study found that the MIC for *S. aureus* and *B. subtilis* was 5 and 4 mg/mL, while the MBC was slightly higher for both species (6.5 and 5 mg/mL). Peng et al. [19] reported stringer anti-staphylococcus activity for patchouli essential oil, which could be due to the different geographical species of the plant, as confirmed by Sufriadi et al. [13].

### 2.2. Physical Properties of Hydrogel Samples

Six samples of hydrogels were prepared using the two pure starches as controls (CS and WS) and four hydrogel mixes, as mentioned in the method section. Table 1 presents the physical characteristics and the appearance of the hydrogel samples. All the samples exhibited deep white color and the controls were odorless, unlike the loaded samples that had the strong unique odor of patchouli essential oil. Due to the very thorough mixing during the preparation, all the samples appeared homogeneous and had a natural pH ranging from 6.81 to 7.23. The physical characteristics of prepared samples indicated their suitability for wound dressing applications [20]. The viscosity of hydrogel is determined by several factors, including the type and concentration of precursor material/s in addition to the crosslinking between the materials. A significant difference in viscosity can be observed between the pure hydrogel samples; conventional corn hydrogel had a viscosity value of only 3973 ± 81 cP compared with waxy starch hydrogel (5321 ± 37 cP). Carbopol was used in this study as a crosslinker and emulsifying agent, which was fixed in all the samples, leaving the concentration of starch playing the main role in hydrogel properties. Sample CWS6 had the highest viscosity of 15,016 ± 59 cP followed by CCS6 with 13,008 ± 29 cP. Interestingly, sample CWS3 had a closely similar viscosity value to CCS6, which can be attributed to the better properties of waxy starch in crosslinking with Carbopol and oil molecules. The spreadability of hydrogel samples ranged from 7.71 ± 0.94 g·cm/s (the control) to 4.02 ± 0.34 g·cm/s in CWS6, which indicates a good spreadability. The low spreadability value in waxy starch hydrogel can be explained by the fact that spreadability is inversely proportion with the viscosity; although sample CWS6 showed the highest viscosity, its spreadability is still highly suitable for wound dressing applications. Loading the starch and essential oil in the hydrogel significantly increased the viscosity and thus limited the spreadability. In fact, high viscosity and medium spreadability in a hydrogel are optimal for wound dressing, which immobilizes the drug for a longer time compared with high spreadability hydrogels [21].

**Figure 2:**
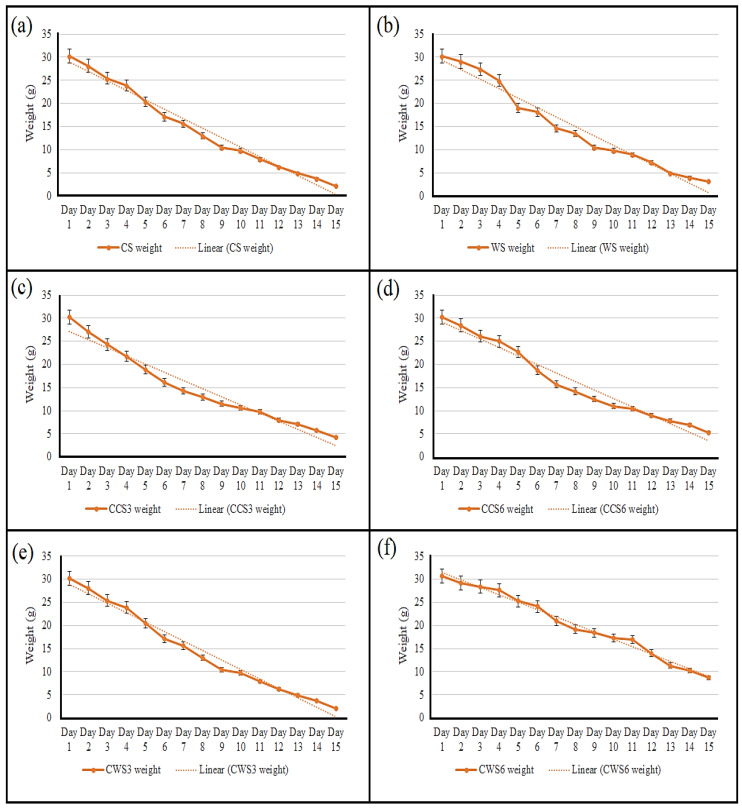
Water evaporation and in-air stability of hydrogel samples; the graphs show the weight reduction in each sample (**a**) CS, (**b**) WS, (**c**) CCS3, (**d**) CCS6, (**e**) CWS3 and (**f**) CWS6 for 15 days.

A water evaporation test was conducted at ambient temperature by placing a certain weight of each sample in open air and monitoring their daily weight reduction for two weeks. Figure 2 presents the water evaporation of all the prepared samples, in which it can be seen that pure starch hydrogels had a faster evaporation rate compared with the loaded samples. CWS6 had the best stability, with reduction in weight from 30.4 g to 7.2 g. Most of the pharmaceutical formulations undergo fast evaporation and, thus, they were kept in closed containers [22]. From Figure 2, the fast evaporation rate and instability of the two controls can be clearly seen; compared with loaded samples, the crosslinking that occurred between starch molecules and Carbopol and between Carbopol and patchouli essential oil enhances the stability of the hydrogel and prevents their water evaporation. This characteristic is very important in wound healing applications, which can preserve a humid environment in the wound and thus provide the optimum conditions for the wound healing process [23]. Placing the formulation in closed containers can significantly enhance the shelf life of the formulation, which is highly desired in the pharmaceutical industry.

### 2.3. Morphological Properties of Hydrogel Samples

**Figure 3:**
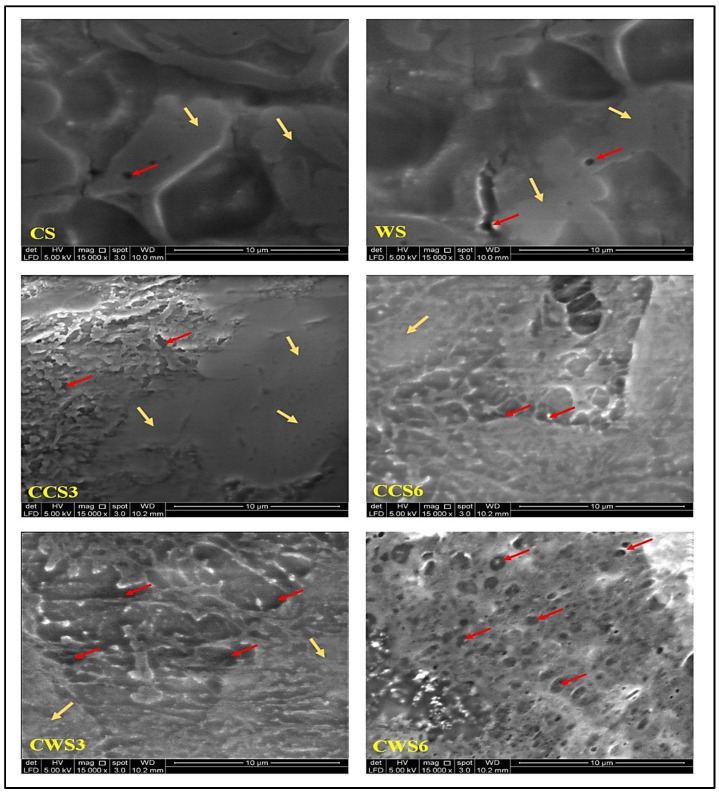
Surface morphology of prepared hydrogel samples under SEM at 15k magnification power; the red arrows show the porous surfaces and the yellow ones show the smooth surfaces.

Figure 3 indicates SEM images of the prepared hydrogel samples, showing the internal network structure and composites of each sample. The lack of networking among the two controls (CS and WS) can be seen compared with loaded samples. The samples of waxy starch have a better porous structure than those with conventional starch. The samples with conventional starch appeared more watery and with less networking compared with the waxy starch ones that showed more particularity and porosity. Similar results for pure starch hydrogels were obtained by [24], who prepared hydrogels from rice bran starch and obtained non porous hydrogels. The lack or limited porosity of the hydrogels could be advantageous, as it prevents the bacterial penetration that may lead to wound infection [25]. Loaded samples showed that the essential oil perfectly mixed with the system in a better way in waxy starch (CWS3 and CWS6) compared with the conventional-based starch (CCS3 and CCS6). The developed hydrogels, with their limited porosity and antibacterial activity, can effectively serve as a barrier for microorganisms.

### 2.4. Fourier Transform Infrared Spectroscopy (FT-IR) Spectra

**Figure 4:**
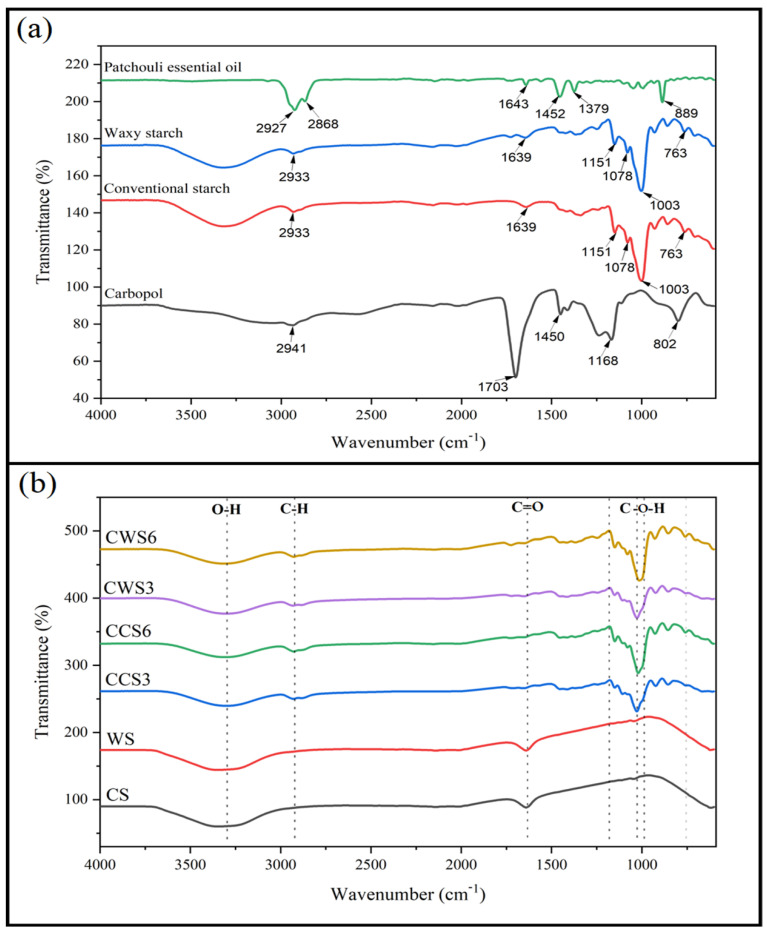
FT-IR spectra of the precursor materials (**a**) and the prepared hydrogel samples (**b**) at a wave number range from 4000 to 500 cm^−1^.

The FT-IR spectroscopy analysis in this study was performed to evaluate the surface functional groups of the precursor materials (Figure 4a), including patchouli essential oil, waxy and conventional starch, in addition to Carbopol and the developed hydrogel samples (Figure 4b), to indicate the existence of particular functional groups in each sample. No significant difference can be observed in the peaks between the waxy starch and the conventional one. However, slight vibration can be seen in the hydrogel’s samples of waxy starch hydrogels, which indicates possible interaction between the three materials. Broad peaks can be clearly observed at around 3300 cm^−1^ in all the hydrogel samples and the two-starch precursors corresponding to the –OH groups. These groups are responsible for the swelling capacity of the hydrogel samples [26]. Thus, loaded samples had slightly altered peaks, but they still exhibiting high swelling capacity. Interestingly, only loaded samples presented sp3 hybridized hydrocarbon chains at 2940 cm^−1^, which confirms the crosslinking between the three materials. The peak in Carbopol spectra at 1703 cm^−1^ corresponds to ketone or aldehyde C=O stretching; this peak is absent in all the loaded samples, due to the crosslinking and the significantly low amount of Carbopol in the samples. However, the peak at 1450 cm^−1^ in Carbopol materials and loaded hydrogels can be clearly observed, which is associated with CH_2_ bending in addition to CH_3_ bending [27].

### 2.5. In Vitro Degradation Study

**Figure 5:**
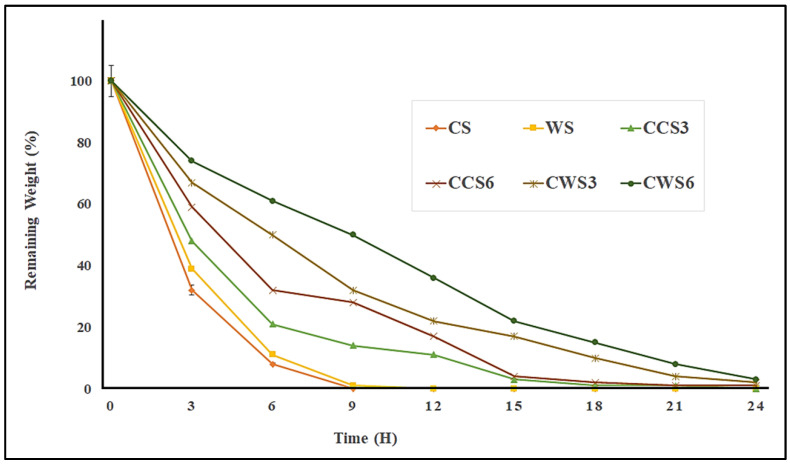
In vitro degradation properties of hydrogel samples in phosphate buffer solutions at 37 °C and pH 7.

The degradation behavior of all the samples in this study was characterized by the percentage of mass loss (Figure 5). After a few hours of soaking the hydrogels in the phosphate buffer solution, the hydrogels degraded and dissolved in different manners. Pure conventional and waxy starch hydrogels had a fast degradation rate, with complete degradation in 9 h. However, loaded samples that contained both the essential oil and the Carbopol exhibited a slower degradation rate. In general, it can be seen that waxy starch samples had a slower rate of degradation, especially CWS6, that remained at 2% of its weight even after 24 h. in wound dressing applications, the hydrogel will be in contact with the wet skin of the wound (and not in the blood), thus, the degradation of our prepared samples will remain for more than 15 days in open air, as described earlier in an evaporation experiment. Wet hydrogels have the advantage of easy spreadability, unlike dried hydrogels, films or aerogels. Although the materials mentioned may possess a longer degradation time, as reported in [28], wet hydrogels are known for their better dressing activity and stronger antibacterial activity [29].

### 2.6. The Rate of Essential Oil Release

**Figure 6:**
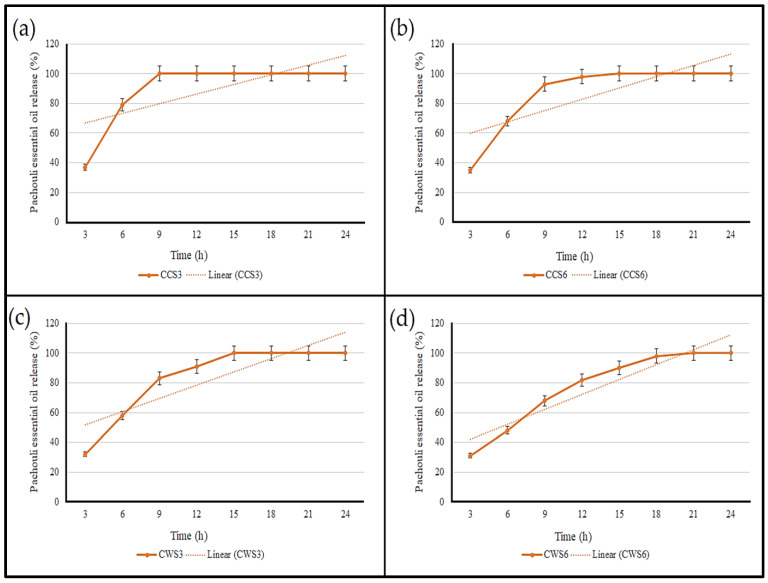
The rate of patchouli essential oil released from loaded hydrogel samples at different time intervals; (**a**) CCS3, (**b**) CCS6, (**c**) CWS3 and (**d**) CWS6.

The release profile of patchouli essential oil from loaded hydrogels into the buffer solution is presented in Figure 6. The pH of the buffer solution was set at 5.5, which is the skin pH as reported in previous studies [30,31]. It can be clearly seen that both conventional starch-based hydrogels exhibited a rapid release of the essential oil, with a complete release within 9 and 12 h for CCS3 and CCS6, respectively. The concentration of the polymeric material/s within the hydrogel has a great impact on the release rate; our study found that samples with 6% starch possessed slower drug release compared with those of 3%, which could be attributed to the change in the specific area resulting in a change in the dissolution rate of the essential oil and its absorption in the hydrogel system [32]. Interestingly, waxy starch-based hydrogels had a sustained and slower release of the essential oil, which lasted for 21 h. This could be attributed to the interaction and chemical bonding between the essential oil, waxy starch and Carbopol, which slowed their release and increased the hydrophobicity of the hydrogel. As a semisolid material, hydrogels are suitable for wound dressing and healing applications; this can fill in the gap resulting from the wound, presenting microbial infection and enhancing the proliferation of cells [23]. The slow release of the essential oil provides sustained antibacterial action, which is highly required in wound treatment.

### 2.7. Antibacterial Activity and Biocompatability of the Hydrogel Samples

**Figure 7:**
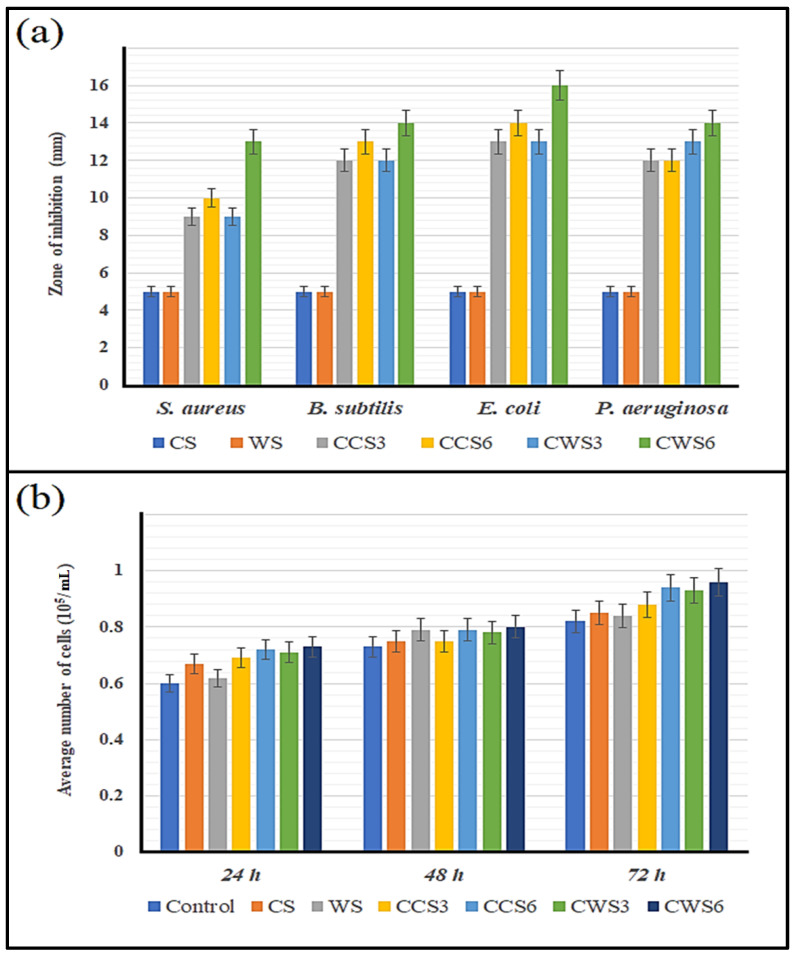
The results of antibacterial activity (**a**) and murine fibroblast biocompatibility (**b**) of prepared hydrogel samples.

The antibacterial activity of the hydrogel samples was assessed in a similar method as the patchouli essential oil, without any dilution of the samples. Figure 7a shows the results of antibacterial activity of hydrogels against the two gram-positive (*S. aureus* and *B. subtilis*) and two gram-negative bacteria (*E. coli* and *P. aeruginosa*). As expected, pure starch hydrogels did not show any antibacterial activity, due to the lack of patchouli essential oil. Interestingly, CWS6 had 10 to 20% higher antibacterial activity than the other samples, which could be explained by the increase in hydrogel viscosity that led to sustained and slow delivery of the essential oil [33]. Although all the samples had the same oil concentration, the high starch content (CCS6 and CWS6) showed better activity, which confirms our hypothesis of sustained delivery. The same pure essential oil earlier had a slightly higher activity against all the bacteria, but incorporating the essential oil in the hydrogel makes it suitable for wound healing, which will prevent its easy evaporation and avoid the use of a toxic solvent to dilute the essential oil. In a previous study, Namazi et al. [26] used oxidized starch hydrogel loaded with ZnO nanoparticles as an antibacterial material. Despite using a chemical material as an antibacterial agent, the authors reported a lower zone of inhibition than that reported in this study. Another study used a mix of essential oils, with a higher portion in the hydrogel and a reported zone of inhibition ranging from 14 to 20 mm [34]. The strong antibacterial activity of patchouli essential oil and the optimal hydrogel mixture promoted our prepared sample for wound healing applications.

To confirm the suitability of the developed hydrogels for medical application, biocompatibility and murine fibroblast proliferation assays were developed. Figure 7b presents the results of the cells’ viability against different samples after a period of incubation for 24, 48 and 72 h. No significant toxicity was observed in all tested samples; even after three days, the samples containing 6% starch showed better cell growth compared with those of 3% and the controls. Surprisingly, no significant difference was also observed between the conventional and waxy starch in terms of cell proliferation, suggesting that the prepared samples are biocompatible and applicable for wound healing and medical applications [35]. Similar findings have been achieved in several plant extracts and other essential oils [36,37]. These studies have reported that the active compounds within essential oil stimulate both the proliferation and migration of fibroblasts, and thus can be used to promote wound healing activity. The activity of patchouli essential oil loaded hydrogels can be the result of the synergistic effects of the associated compounds present, such as α and β-Patchoulene, Patchoulol, Pogostol and patchouli alcohol, which is reported in the literature as possessing potential anti-inflammatory and antimicrobial activity [38]. In a similar study conducted by Seyed Ahmadi et al. [39], the authors reported that cinnamon essential oil enhances the release of insulin-like growth factor 1 (IGF-1), which also participates in the wound healing process by enhancing the proliferation of fibroblast cells. Our data showed an increase in cell proliferation when treated with patchouli essential oil in a similar manner reported in this study, which can also be attributed to IGF-1 expression. This study has clearly demonstrated that patchouli essential oil loaded starch hydrogels possessed good antibacterial activity against both gram-positive and -negative bacteria. Furthermore, the samples were totally biocompatible and enhanced the proliferation of murine fibroblast, which indicates their suitability for further animal and clinical trial studies.

## 3. Conclusions

In this work, conventional and waxy corn starch/Carbopol hydrogels were successfully fabricated and loaded with patchouli essential oil. The viscosity and spreadability of hydrogel were highly affected by the type and concentration of starch. Waxy starch was found to better mix with both patchouli essential oil and Carbopol, producing higher porosity hydrogel and enhanced viscosity. The extracted patchouli essential oil exhibited high antibacterial activity against all selected strains. Owing to the unique active compounds in patchouli essential oil and its high antioxidant activity, loaded samples possessed good antibacterial activity and higher biocompatibility. The presence of sp3 hybridized hydrocarbon chains at 2940 cm^−1^ confirms the homogeneous crosslinking between the three materials. Overall, the merits of good antibacterial activity and enhanced biocompatibility make these formulations promising biomedical candidates for wound healing applications.

## 4. Materials and Methods

### 4.1. Materials

In this study, *Pogostemon cablin* (Patchouli) were purchased from Aceh Jaya, 24784 Banda Aceh, Indonesia. Patchouli essential oil was extracted in the lab using a supercritical carbon dioxide (ScCO_2_) hydro-distillation approach via a Clevenger apparatus. Conventional corn and waxy starches were obtained from Agricore CS Sdn Bhd (Penang, Malaysia). Other chemicals, including Carbopol, glycerol and triethanolamine, were supplied by Sigma Aldrich (Selangor, Malaysia).

### 4.2. Extraction of Patchouli Essential Oil

Patchouli essential oil was extracted from dried patchouli leaves using the Sc CO_2_ fluid extraction apparatus in the same method described in [40], with the following modifications: the temperature was increased to 80 °C and 10 MPa pressure to enhance the extraction performance. The extracted patchouli essential oil was hydro-distillated for 4 h in a circulatory Clevenger apparatus and then characterized by FT-IR and antibacterial activity.

### 4.3. Preparation of Loaded and Non-Loaded Hydrogels

Table 2 presents the chemical composition of each hydrogel sample. They were prepared by mixing two phases, the starch phase and the Carbopol phase, together, and then loading them with the essential oil. A similar preparation was employed for both types of starch: 3 and 6% solution of each type was heated at 90 °C for 1 h with constant stirring to plasticize the starch molecules. On the other hand, 0.25% solution of Carbopol was prepared and stirred for 4 h until a clear solution was formed. Similar amounts of starch and Carbopol were mixed together and stirred for a few minutes until a homogenized solution was formed. Then, 3 mL of Patchouli essential oil was dissolved in 5 mL of glycerol and slowly added to the mixture with continuous stirring for 30 min. Finally, to adjust the viscosity and pH, 0.05 mL of triethanolamine was added as a cross linker for the Carbopol, and thick hydrogels were formed. A hydrogel of conventional corn and waxy starches without patchouli essential oil was also prepared as a control sample.

### 4.4. Antibacterial Activity, MIC and MBC of Patchouli Essential Oil

The disk diffusion method was applied for determining the antibacterial activity of the essential oil, as described in [41]. Four microorganisms, namely *Staphylococcus aureus*, *Bacillus subtilis*, *Escherichia coli* and *Pseudomonas aeruginosa*, were used along with dimethyl sulfoxide for the dilutions of the essential oil. The minimum inhibitory concentration (MIC) for the essential oil against each type of bacteria was calculated by using the serial dilution approach and monitoring the bacterial growth in the Mueller–Hinton broth media. Minimum bactericidal concentration (MBC) was then determined by taking 20 μL from the clear tubes of the MICs and inoculating them on a fresh nutrient agar media.

### 4.5. Characterization of Hydrogels

#### 4.5.1. Physical and Morphological Analysis

A black background was used to determine the color of the hydrogel samples; the odor was determined by mixing each sample in distilled water and taking in the smell. A digital pH meter (HANNA Ltd., Eden Way, Pages Industrial Park, Leighton Buzzard, Bedfordshire LU7 4AD, UK) was for pH measurement, while a Brookfield viscometer (model DV-11+ Pro, Mecomb Malaysia Sdn Bhd, Puchong Utama 47100 Puchong, Selangor) measured the viscosity using a spindle rotating in room temperature and at 50 RPM. The spreadability of the prepared hydrogels was also evaluated by measuring the spreadability diameter of 1 g of each sample between two glass plates in the same method described in [42]. The surface morphology was examined by Scanning Electron Microscope with a high resolution (Carl Zeiss Group, Oberkochen, Germany).

#### 4.5.2. Surface Functional Groups

The surface functional groups of patchouli essential oil and hydrogels were investigated by a FT-IR spectrum (RAffinity-1S equipped with an AIM-9000, Shimadzu Corp. Ltd., Kyoto, Japan) using the attenuated total re-308 flection (ATR) approach in the wavenumber range of 4000–400 cm^−1^.

#### 4.5.3. Antibacterial Activity

The antibacterial activity of all the prepared hydrogel samples was determined by the disk diffusion method in the same approach as the patchouli essential oil. The experiment was performed three times and the mean value was taken as the result.

#### 4.5.4. In Vitro Biodegradation

The biodegradation behavior of all prepared hydrogels was evaluated in a wet state in the same method described in [43], with the following modifications. A known weight of each sample was placed in a sealed tube containing phosphate buffer solutions (pH = 7.4) at 37 °C. After that, the hydrogels were removed and weighted at regular intervals after wiping the surface water with filter paper. This process was repeated until the hydrogels were completely biodegraded.

#### 4.5.5. In Vitro Essential Oil Release

The essential oils released from the prepared hydrogels were carried out in the same method described by Quintanilla de Stéfano et al. [44]. In brief, a known weight of each loaded hydrogel was transferred into phosphate buffer solutions of pH 5.5 (skin pH) and ambient temperature. At specific time intervals, the hydrogel samples of the buffer solution were withdrawn and the concentration of the released essential oil in the buffer solution was determined by UV-Vis spectrophotometer (Shimadzu Corp. Ltd., Kyoto, Japan). All experiments were triplicated for each sample and the average value was considered as the result. The patchouli essential oil’s release percentage was finally calculated using the following equation:

$$\mathrm{Patchouli}\;\mathrm{essential}\;\mathrm{oil}\;\mathrm{release}\;\left(\%\right)=\left(\frac{\mathrm{Mt}}{\mathrm{Mo}}-1\right)\times 100$$

where Mo is the initial mass of patchouli essential oil loaded in the hydrogel and Mt is the mass of the essential oil released at time t.

#### 4.5.6. Biocompatibility Assay

Biocompatibility experiments of all the prepared samples were performed by measuring the proliferation of murine fibroblast (L929, ATCC) in the same approach described by Villamizar-Sarmiento et al. [45], using 0.3 × 10^5^ cells/mL and an incubation condition of 5% (*v*/*v*) CO_2_ at 37 °C for 24 and 48 h.

## Figures and Tables

**Table 1:** Physical appearance and properties of prepared hydrogel samples.

Sample	Physical Appearance and Odor	Homogeneity	pH	Spreadability (g·cm/s)	Viscosity (cP)	Hydrogel Photograph
CS	Deep white and odorless	Watery	6.81 ± 0.2	7.71 ± 0.94	3973 ± 81	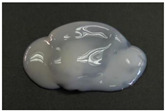
WS	Deep white and odorless	Watery	6.97 ± 0.2	6.94 ± 0.82	5321 ± 37	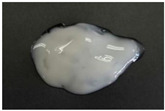
CCS3	Deep white with strong patchouli smell	Good	7.13 ± 0.1	5.26 ± 0.64	10,917 ± 82	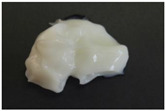
CCS6	Deep white with strong patchouli smell	Excellent	7.04 ± 0.2	4.59 ± 0.88	13,008 ± 29	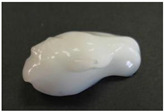
CWS3	Deep white with strong patchouli smell	Good	7.23 ± 0.1	4.87 ± 0.71	12,411 ± 91	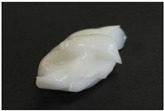
CWS6	Deep white with strong patchouli smell	Excellent	7.19 ± 0.1	4.02 ± 0.34	15,016 ± 59	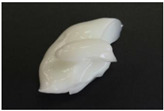

**Table 2:** Mixture design and chemical composition of hydrogel samples.

Sample	Conventional Starch (%)	Waxy Starch (%)	Carbopol (%)	Patchouli Essential Oil (%)	Glycerol (%)	Triethanol-Amine	Water (%)
CS	6	-	-	-	-	-	94
WS	-	6	-	-	-	-	94
CCS3	3	-	0.25	3	5	0.05	88.7
CCS6	6	-	0.25	3	5	0.05	85.7
CWS3	-	3	0.25	3	5	0.05	88.7
CWS6	-	6	0.25	3	5	0.05	85.7

## Data Availability

Not applicable.
